# Serum sodium trajectories impact clinical outcomes in extracorporeal resuscitation of cardiac arrest: A multicenter retrospective cohort study

**DOI:** 10.1016/j.resplu.2025.101074

**Published:** 2025-08-22

**Authors:** Yu Amemiya, Ryo Hisamune, Kazuma Yamakawa, Ryosuke Zushi, Hitoshi Kobata, Akihiko Inoue, Toru Hifumi, Tetsuya Sakamoto, Yasuhiro Kuroda, Akira Takasu, the SAVE-J II Study Group, Hirotaka Sawano, Yuko Egawa, Shunichi Kato, Naofumi Bunya, Takehiko Kasai, Shinichi Ijuin, Shinichi Nakayama, Jun Kanda, Seiya Kanou, Toru Takiguchi, Shoji Yokobori, Hiroaki Takada, Kazushige Inoue, Ichiro Takeuchi, Hiroshi Honzawa, Makoto Kobayashi, Tomohiro Hamagami, Wataru Takayama, Yasuhiro Otomo, Kunihiko Maekawa, Takafumi Shimizu, Satoshi Nara, Michitaka Nasu, Kuniko Takahashi, Yoshihiro Hagiwara, Shigeki Kushimoto, Reo Fukuda, Takayuki Ogura, Shin-ichiro Shiraishi, Hiroshi Okamoto, Norio Otani, Migaku Kikuchi, Kazuhiro Watanabe, Takuo Nakagami, Tomohisa Shoko, Nobuya Kitamura, Takayuki Otani, Yoshinori Matsuoka, Makoto Aoki, Masaaki Sakuraya, Hideki Arimoto, Koichiro Homma, Hiromichi Naito, Shunichiro Nakao, Jun Kunikata, Hideto Yokoi, Tomoya Okazaki, Yoshio Tahara

**Affiliations:** h Osaka Saiseikai Senri Hospital, Japan; i Saitama Red Cross Hospital, Japan; j Sapporo Medical University, Japan; k Hyogo Emergency Medical Center, Japan; l Teikyo University Hospital, Japan; m Nippon Medical School, Japan; n National Hospital Organization Disaster Medical Center, Japan; o Yokohama City University Medical Center, Japan; p Toyooka Public Hospital, Japan; q Tokyo Medical and Dental University Hospital of Medicine, Japan; r Hokkaido University Hospital, Japan; s Teine Keijinkai Hospital, Japan; t Urasoe General Hospital, Japan; u Imperial Foundation Saiseikai Utsunomiya Hospital, Japan; v Tohoku University Graduate School of Medicine, Japan; w Nippon Medical School Tama Nagayama Hospital, Japan; x Japan Red Cross Maebashi Hospital, Japan; y Aizu Central Hospital, Japan; z St. Luke’s International Hospital, Japan; aa Dokkyo Medical University, Japan; ab Nihon University Hospital, Japan; ac Omihachiman Community Medical Center, Japan; ad Tokyo Women’s Medical University Medical Center East, Japan; ae Kimitsu Chuo Hospital, Japan; af Hiroshima City Hiroshima Citizens Hospital, Japan; ag Kobe City Medical Center General Hospital, Japan; ah Gunma University Graduate School of Medicine, Japan; ai JA Hiroshima General Hospital, Japan; aj Osaka City General Hospital, Japan; ak Keio University School of Medicine, Japan; al Okayama University Hospital, Japan; am Osaka University Graduate School of Medicine, Japan; an Kagawa University Hospital, Japan; ao National Cerebral and Cardiovascular Center, Japan; a Department of Emergency and Critical Care Medicine, Osaka Medical and Pharmaceutical University, 2-7 Daigakumachi, Takatsuki, Japan; b Department of Cardiology, Osaka Medical and Pharmaceutical University, 2-7 Daigakumachi, Takatsuki, Japan; c Department of Neurosurgery and Emergency Medicine, Tane General Hospital, 1-12-21 Kujo-minami, Nishi-ku, Osaka, Japan; d Department of Emergency and Critical Care Medicine, Hyogo Emergency Medical Center, 1-3-1 Chuo-ku, Kobe, Hyogo, Japan; e Department of Emergency and Critical Care Medicine, St. Luke’s International Hospital, 9-1 Akashi-cho, Chuo-ku, Tokyo, Japan; f Department of Emergency Medicine, Teikyo University School of Medicine, 2-11-1 Kaga, Itabashi-ku, Tokyo, Japan; g Department of Emergency Medicine, Kagawa University School of Medicine, 1750-1 Ikenobe, Miki-cho, Kita-gun, Kagawa, Japan

**Keywords:** Cardiac arrest, Extracorporeal membrane oxygenation, Hypernatremia, Hyponatremia, Trajectory, Clustering

## Abstract

**Background:**

Dysregulation of serum sodium levels subsequent to out-of-hospital cardiac arrest (OHCA) has the potential to influence neurological outcomes. The patterns of sodium correction remain inadequately studied in individuals undergoing extracorporeal cardiopulmonary resuscitation (ECPR). This investigation aimed to elucidate the correlation between the trajectories of serum sodium concentration and clinical outcomes in patients subjected to ECPR.

**Methods:**

This study was a secondary analysis of the SAVE-J II study, a retrospective multicenter registry targeting OHCA adult patients resuscitated with extracorporeal membrane oxygenation in Japan. Using the k-means clustering method, patients were categorized into four clinically relevant clusters: normal range, corrected hyponatremia, overcorrected, and high trend. The primary outcome was a favorable neurological outcome at 30 days, and the secondary outcome was survival at hospital discharge.

**Results:**

Among the 400 patients who were resuscitated with extracorporeal membrane oxygenation, 170 (42.5 %) had favorable neurological outcomes, and 305 (76.3 %) survived to discharge. Multivariable analysis revealed that younger age (OR: 0.97, 95 % CI: 0.95–0.99), bystander cardiopulmonary resuscitation (OR: 1.92, 95 % CI: 1.19–3.14), and transient return of spontaneous circulation (OR: 1.89, 95 % CI: 1.01–3.55) were associated with favorable neurological outcomes. No significant differences in neurological outcomes were observed between the clusters. Patients in the overcorrected cluster demonstrated significantly lower odds of survival at discharge (OR: 0.33, 95 % CI: 0.15–0.73).

**Conclusions:**

While overcorrection of serum sodium levels in OHCA patients receiving ECPR was not associated with neurological outcomes, it was independently associated with decreased survival to hospital discharge.

## Highlights

• Four distinct sodium trajectories identified in OHCA patients receiving ECPR.
• K-means clustering revealed clinically meaningful sodium correction phenotypes.
• Overcorrection pattern independently reduced survival odds by 20 % vs controls.
• No significant association found between sodium patterns and neurologic outcomes.
• Gradual sodium management may improve survival in ECPR patients.

## Graphical abstract

**Figure:**
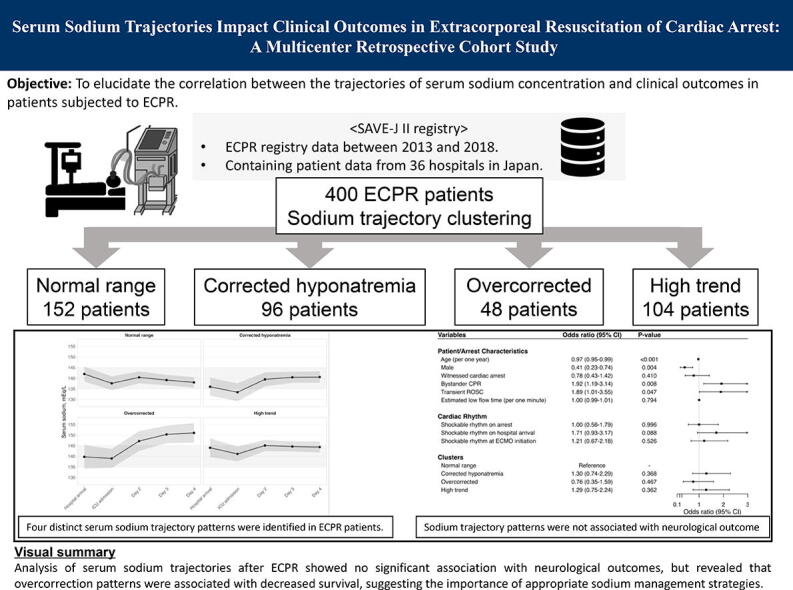

## Introduction

Out-of-hospital cardiac arrest (OHCA) is experienced by nearly 4 million individuals worldwide each year, with fewer than 10 % of adults surviving with good neurologic recovery, despite improvements in resuscitation practices.^1^ According to emerging evidence in recent studies, extracorporeal cardiopulmonary resuscitation (ECPR) may offer potential survival benefits even after conventional resuscitation has failed.^2^, ^3^, ^4^ However, recent European data on ECPR revealed that only 19 % of ECPR patients achieve favorable neurological outcomes at 3 months, and favorable neurological outcomes are observed in only 9 % of OHCA patients.^5^ These outcomes, while representing lives saved that would otherwise be lost, underscore the substantial room for improvement in ECPR implementation and patient selection.

In patients resuscitated from OHCA, dysregulation of serum sodium levels has been associated with unfavorable neurological outcomes. Previous studies have demonstrated that both hypernatremia and hyponatremia during the postcardiac arrest period may adversely affect neurological recovery through distinct pathophysiological mechanisms.^6^, ^7^ Hypernatremia can trigger water shifts from neuronal cells in the brain, leading to cellular dehydration and shrinkage.^8^ In contrast, hyponatremia may cause excessive water movement into brain cells, resulting in cellular edema and increased intracranial pressure.^9^ The integrity of the blood–brain barrier (BBB) is known to be compromised for at least 72 h following cardiac arrest.^10^ This BBB disruption results from a complex interplay of ischemia–reperfusion injury,^10^ inflammation,^11^ and edema formation. Furthermore, experimental studies by Olson et al. have suggested that hyponatremia itself may increase BBB permeability and cerebral blood flow, potentially contributing to cerebral edema formation.^12^

No study has analyzed the temporal patterns of sodium changes throughout the period of BBB vulnerability. On the basis of experimental evidence that hyponatremia increases BBB permeability and contributes to cerebral edema formation, we hypothesized that sustained or uncorrected hyponatremia during the initial 72-h period would adversely affect neurological recovery. Conversely, we also hypothesized that mild hypernatremia might confer neuroprotective effects by reducing cellular edema and maintaining osmotic gradients favorable for cerebral perfusion. We therefore investigated whether different sodium correction trajectories, characterized by both absolute levels and temporal change patterns, are correlated with varying outcomes in postcardiac arrest patients resuscitated with extracorporeal membrane oxygenation (ECMO).

## Material and methods

### Study design and setting

This study was a secondary analysis of the SAVE-J II study,^13^ a retrospective multicenter registry study of OHCA patients resuscitated with ECMO, involving 36 participating institutions in Japan. The SAVE-J II study included consecutive OHCA patients of 18 years of age and older who were resuscitated with ECMO and admitted to the participating institutions between January 1, 2013, and December 31, 2018.

This study was conducted in accordance with the ethical principles of the Declaration of Helsinki and approved by the Clinical Research Ethics Committee of Kagawa University (Approval Number: 2018-110) as well as by the ethics committees of all participating institutions. Owing to the retrospective nature of the study, the requirement of informed consent was waived at all participating facilities.

### Study population

All patients enrolled in the SAVE-J II study were initially considered for this secondary analysis. Patients were excluded if they experienced cardiac arrest in the hospital, had return of spontaneous circulation (ROSC) at ECMO initiation, had unknown outcomes or diagnoses, had a “do not attempt resuscitation” (DNAR) order, died within the first 4 days of intensive care unit (ICU) admission, or had any missing serum sodium values on arrival at the hospital and on ICU admission to day 4. Unknown diagnoses were defined as cases for which the cause of cardiac arrest could not be classified into established categories including cardiac diseases, cerebral disorders, drug intoxication, pulmonary embolism, external causes, trauma, or other identifiable etiologies.

Time series data of serum sodium levels from hospital arrival through ICU day 4 were analyzed. Patients were classified into four clinically relevant clusters: a normal-range (NR) cluster, a corrected-hyponatremia (CH) cluster, an overcorrected (OC) cluster, and a high-trend (HT) cluster.

### Data collection

The following data were collected from the SAVE-J II registry: demographic characteristics (age, sex), cardiac arrest circumstances (witnessed arrest, bystander-initiated cardiopulmonary resuscitation (CPR), location), cardiac rhythm, treatment factors (adrenaline administration, defibrillation, prehospital airway management), cause of cardiac arrest, and ECMO parameters. Serum sodium levels were measured at five specific time points: at hospital arrival, at ICU admission, and consecutively on ICU days 2, 3, and 4 after admission. The following additional data were collected: length of hospital and ICU stays, presence of acute kidney injury, requirement for renal replacement therapy, duration of mechanical ventilation, and in-hospital mortality. The estimated low-flow time was calculated as the duration from cardiac arrest to the establishment of ECMO. ECPR refers to resuscitation via ECMO for patients with refractory cardiac arrest. ROSC was defined as any palpable pulse or measurable blood pressure lasting one minute or more. Transient ROSC indicates the presence of either prehospital ROSC or a detectable waveform during prehospital transportation or at hospital arrival. A shockable rhythm corresponds to ventricular fibrillation or pulseless ventricular tachycardia.

### Outcome measures

The primary outcome was a favorable neurological outcome, defined as a cerebral performance category (CPC) score of 1 or 2 at 30 days. The survival rate at hospital discharge was assessed as a secondary outcome.

### Statistical analysis

For cluster analysis of sodium level trajectories, we employed the k-means algorithm applied to a two-dimensional parameter space. Each patient was characterized by two parameters extracted from their time series data: (1) the mean serum sodium level across the observation period (hospital arrival through ICU day 4); and (2) the slope of the change in sodium concentration calculated via linear regression (Supplementary Fig. S1). The two-dimensional parameter space reflects clinical sodium management practices, whereby physicians routinely perform daily monitoring and correction toward normal values in critically ill patients. In clinical settings, therapeutic interventions are guided by assessment of both the absolute sodium level and the trajectory of change over time. This dual approach addresses fundamental clinical considerations: baseline severity and temporal patterns of sodium change, which together guide treatment intensity. The mean sodium level represents overall sodium disorder severity during the critical period, whereas the slope captures the dynamic response to therapeutic interventions or underlying disease progression. Four clusters were selected using the elbow method (Supplementary Fig. S2) based on statistical adequacy and clinical interpretability.

Multivariable logistic regression analysis was performed to evaluate associations between sodium correction patterns and outcomes, adjusting for previously described covariates, including age, sex, witness status, bystander CPR, cardiac rhythm,^14^ presence of transient ROSC, and estimated low-flow time.^13^, ^15^, ^16^, ^17^, ^18^

To ensure the robustness of our findings, we conducted two sensitivity analyses. First, we performed an alternative analysis excluding patients who died within the first 2 days of ICU admission, instead of the 4-day exclusion criterion used in the primary analysis. Second, missing data were handled using multiple imputation with the random forest algorithm, a non-parametric random forest-based method that accommodates mixed-type data without distributional assumptions. Imputation was performed sequentially following the clinical timeline: baseline demographics and vital signs, hospital arrival laboratory values, ICU admission measurements, and consecutive ICU days 2–4 electrolyte values, with previously imputed values serving as predictors for subsequent time points. The algorithm was configured with 10 iterations and 100 trees per forest. Comprehensive quality assessment demonstrated excellent preservation of distributional properties, correlation structures, and physiological plausibility (Supplementary information). Both sensitivity analyses employed the same clustering and regression methods as the primary analysis.

Continuous variables are presented as medians with interquartile ranges, as appropriate. Categorical variables are presented as counts and percentages. Between-cluster comparisons were performed using the Kruskal–Wallis test for continuous variables and Fisher’s exact test for categorical variables. *P* values of <0.05 were considered to indicate statistical significance. All analyses were performed using R (ver. 4.4.1, R Foundation for Statistical Computing, Vienna, Austria, 2024).

## Results

### Study population

**Fig. 1:**
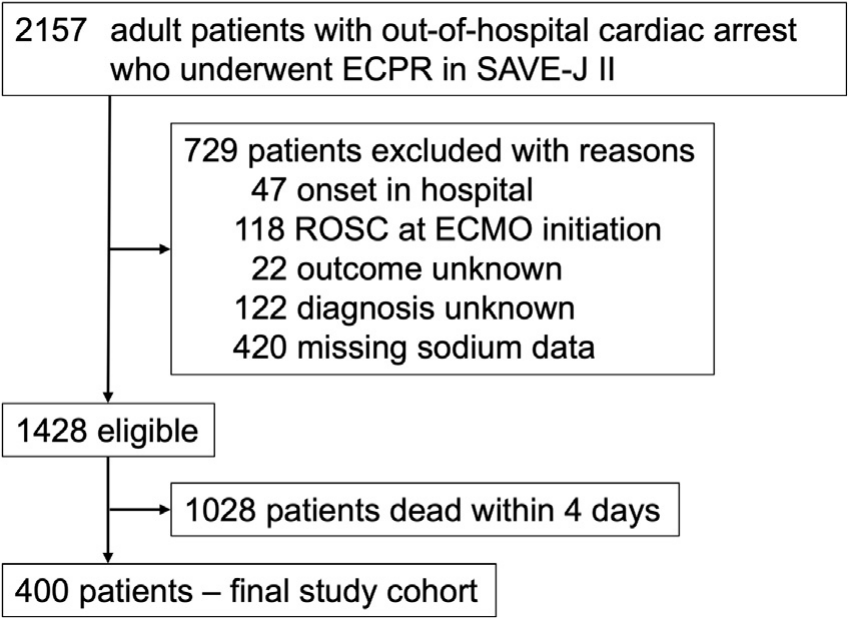
Flowchart of the enrollment of study participants. ECPR, extracorporeal cardiopulmonary resuscitation; ROSC, return of spontaneous circulation; ECMO, extracorporeal membrane oxygenation.

The study population consisted of adult patients with OHCA who underwent ECPR in the SAVE-J II study. From the initial 2,157 patients, we applied the exclusion criteria shown in Fig. 1. Consequently, 400 patients with complete sodium measurements were included in the final analysis.

The median age of the patients was 59 years (interquartile range: 47–67 years). Among these patients, 170 had favorable neurological outcomes, and 305 patients achieved survival to hospital discharge. The median length of ICU stay was 12 days (8–18 days). Patients required mechanical ventilation for a median of 10 days (7–16 days). Active withdrawal of treatment was implemented in 12 patients (Table 1).

**Table 1:** Baseline characteristics by cluster.

Characteristics	NR (*N* = 152)	CH (*N* = 96)	OC (*N* = 48)	HT (*N* = 104)	*P*-value
Age, years	55.0 [42.0, 66.0]	61.0 [51.5, 69.0]	58.5 [46.8, 69.3]	60.0 [51.0, 67.0]	0.006
Male	124 [81.6 %]	84 [87.5 %]	35 [72.9 %]	89 [85.6 %]	0.136
Comorbidities	104 [69.3 %]	72 [78.3 %]	37 [78.7 %]	72 [70.6 %]	0.336
Hypertension	47 [30.9 %]	25 [26.0 %]	12 [25.0 %]	32 [30.8 %]	0.750
Diabetic mellitus	18 [11.8 %]	27 [28.1 %]	11 [22.9 %]	19 [18.3 %]	0.012
Hyperlipidemia	26 [17.1 %]	10 [10.4 %]	5 [10.4 %]	14 [13.5 %]	0.424
Heart disease	37 [24.3 %]	22 [22.9 %]	10 [20.8 %]	19 [18.3 %]	0.702
Cerebral disorder	10 [6.6 %]	3 [3.1 %]	4 [8.3 %]	4 [3.8 %]	0.436
Chronic renal failure	6 [3.9 %]	8 [8.3 %]	1 [2.1 %]	1 [1.0 %]	0.064
Location of cardiac arrest	−	−	−	−	−
Home	43 [28.3 %]	16 [16.7 %]	12 [25.0 %]	22 [21.2 %]	0.033
Public	73 [48.0 %]	50 [52.1 %]	16 [33.3 %]	48 [46.2 %]	0.146
Cardiac arrest witnessed by EMS	16 [10.5 %]	15 [15.6 %]	9 [18.8 %]	19 [18.3 %]	0.234
Witnessed cardiac arrest	125 [82.2 %]	72 [75.0 %]	40 [83.3 %]	87 [83.7 %]	0.385
Bystander cardiopulmonary resuscitation	86 [56.6 %]	61 [63.5 %]	37 [77.1 %]	62 [59.6 %]	0.078
Cardiac rhythm	−	−	−	−	−
ROSC before hospital arrival	24 [15.9 %]	18 [19.4 %]	5 [10.6 %]	15 [14.9 %]	0.595
Shockable rhythm on arrest	123 [80.9 %]	65 [67.7 %]	24 [50.0 %]	74 [71.2 %]	<0.001
Shockable rhythm on hospital arrival	89 [58.6 %]	53 [55.2 %]	20 [41.7 %]	64 [61.5 %]	0.127
Shockable rhythm at ECMO initiation	107 [70.4 %]	62 [64.6 %]	23 [47.9 %]	71 [68.3 %]	0.035
ROSC at hospital arrival	11 [7.2 %]	7 [7.3 %]	3 [6.3 %]	5 [4.8 %]	0.882
Transient ROSC	25 [16.6 %]	19 [20.4 %]	6 [12.8 %]	16 [15.7 %]	0.677
Time course, minutes	−	−	−	−	−
EMS call to hospital arrival	30.0 [24.0, 37.0]	32.0 [25.5, 39.0]	29.5 [25.8, 34.3]	30.0 [26.0, 37.0]	0.559
Hospital arrival to ECMO initiation	21.0 [15.0, 29.5]	20.0 [13.5, 31.0]	20.5 [14.0, 26.5]	21.0 [14.0, 30.0]	0.964
EMS call to ECMO initiation	53.0 [43.0, 63.0]	57.0 [43.5, 72.0]	53.0 [45.0, 62.3]	55.0 [44.0, 66.0]	0.470
Estimated low flow time	52.0 [42.0, 62.3]	52.0 [41.0, 70.5]	50.0 [39.5, 57.3]	50.0 [39.0, 63.0]	0.271
Diagnosis	−	−	−	−	−
Cardiac	−	−	−	−	−
Acute coronary syndrome	87 [57.2 %]	59 [61.5 %]	21 [43.8 %]	60 [57.7 %]	0.239
Arrythmia	28 [18.4 %]	8 [8.3 %]	8 [16.7 %]	12 [11.5 %]	0.086
Myopathy	11 [7.2 %]	5 [5.2 %]	2 [4.2 %]	6 [5.8 %]	0.942
Other cardiac etiologies	23 [15.1 %]	8 [8.3 %]	7 [14.5 %]	7 [6.7 %]	0.158
Acute aortic dissection or aortic aneurysm	0 [0.0 %]	1 [1.0 %]	0 [0.0 %]	2 [1.9 %]	0.243
Pulmonary embolism	4 [2.6 %]	3 [3.1 %]	7 [14.6 %]	8 [7.7 %]	0.011
External	10 [6.6 %]	15 [15.5 %]	5 [10.4 %]	12 [11.6 %]	0.189
After admission status	−	−	−	−	−
Acute kidney injury	56 [37.6 %]	39 [41.9 %]	27 [56.3 %]	32 [31.7 %]	0.034
Renal replacement therapy	38 [25.5 %]	21 [22.6 %]	7 [14.6 %]	17 [17.0 %]	0.255
Water balance in day 3, mL	7,715.0 [5,165.0, 10,312.0]	8,294.0 [5,306.0, 10,638.0]	7,619.0 [4,712.0, 10,445.2]	7,861.0 [4,101.0, 11,119.0]	0.567
Outcomes	−	−	−	−	−
Favorable neurological outcome	65 [42.8 %]	43 [44.8 %]	15 [31.3 %]	47 [45.2 %]	0.393
Hospital discharge survival	122 [80.3 %]	75 [78.1 %]	27 [56.3 %]	81 [77.9 %]	0.011

Data are expressed as percent or median with interquartile range, as indicated.

NR, normal-range cluster; CH, corrected-hyponatremia cluster; OC, overcorrected cluster; HT, high-trend cluster; EMS, emergency medical service; ROSC, return of spontaneous circulation; ECMO, extracorporeal membrane oxygenation.

### Sodium characteristics and clusters

**Fig. 2:**
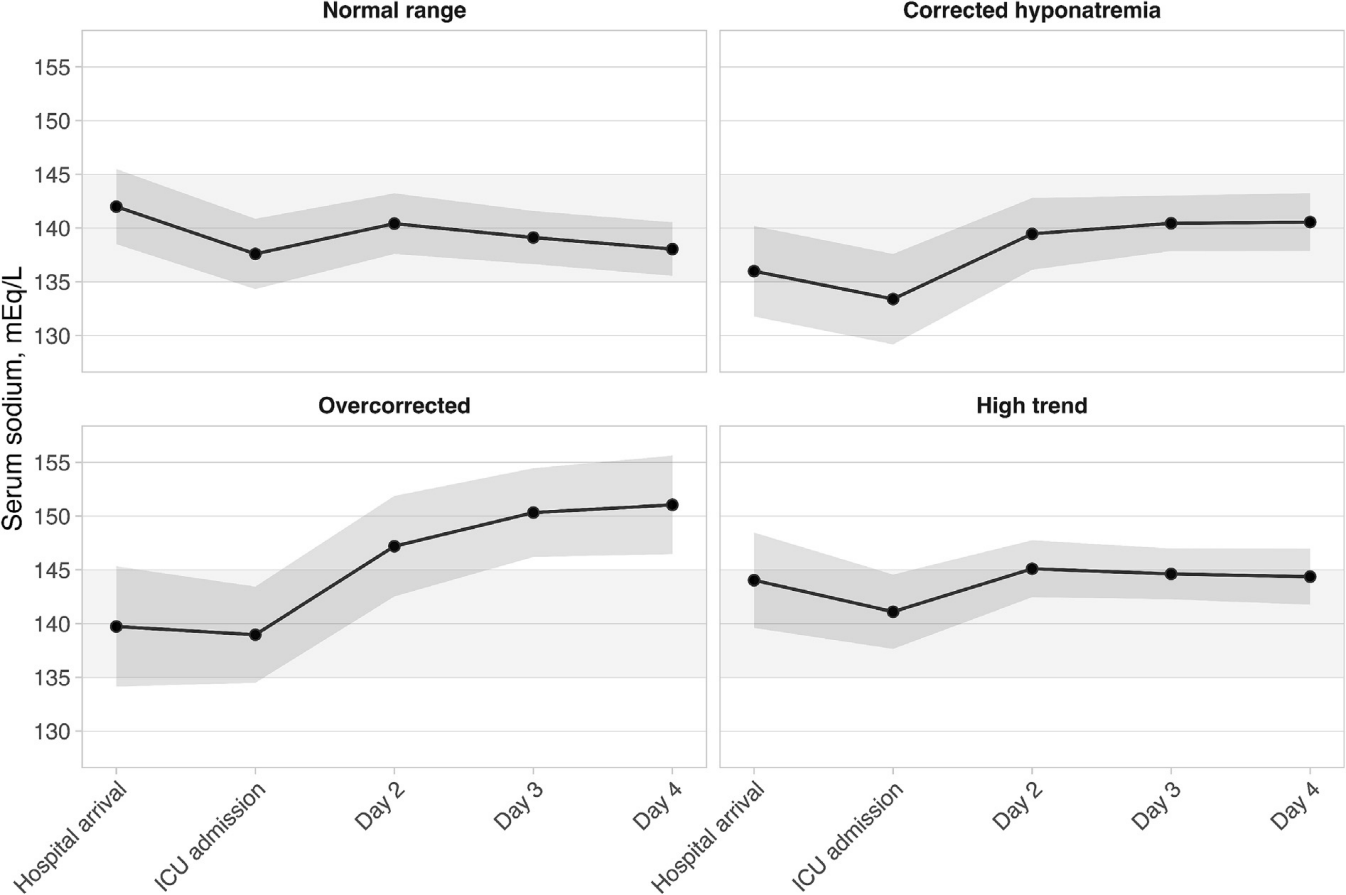
Four distinct trajectories of the serum sodium concentration. Points represent the mean, gray bands represent the normal range of the serum sodium concentration (135–145 mEq/L). Shaded areas represent the standard deviation.

Each cluster exhibited distinctive sodium trajectories over the monitoring period shown in Fig. 2. The CH cluster showed initial normal sodium levels that decreased upon ICU admission, followed by correction to the normal range by day 2. The NR cluster maintained the most stable sodium profile among the normal parameters throughout the observation period. The OC cluster exhibited the most dramatic fluctuations, starting from normal levels but rapidly increasing to hypernatremia values by day 2 and continuing to rise through days 3–4. The HT cluster presented elevated sodium levels at hospital arrival, which remained consistently high throughout the monitoring period. Four distinct sodium trajectory clusters were identified through k-means analysis, resulting in subgroups with characteristic sodium profiles (Supplementary Table S1).

### Baseline characteristics of sodium clusters

The baseline characteristics across these clusters are summarized in Table 1. The NR cluster was the youngest, with a median age of 55 years. The initial shockable rhythm at arrest showed significant differences among the clusters, being highest in the NR cluster (80.9 %) and lowest in the OC cluster (50.0 %). The time interval from the emergency call to treatment was comparable across the clusters.

### Favorable neurological outcome

Multivariable logistic regression analysis revealed several patient factors that were significantly associated with favorable neurological outcomes, including younger age (OR: 0.97; 95 % CI: 0.95–0.99), bystander CPR (OR: 1.92, 95 % CI: 1.19–3.14), and transient ROSC (OR: 1.89, 95 % CI: 1.01–3.55). Male sex was associated with worse neurological outcomes (OR: 0.41, 95 % CI: 0.23–0.74). When the sodium trajectory patterns were examined, no statistically significant differences in neurological outcomes were observed.

### Survival at discharge

Patients in the OC cluster demonstrated significantly lower odds of survival at discharge than those in the NR reference cluster (OR: 0.33, 95 % CI: 0.15–0.73). In comparison to the reference cluster, the CH and HT clusters showed no significant differences in survival (CH, 1.11, 0.56–2.25; HT, 0.96, 0.50–1.88) (Table 2). A forest plot of odds ratios and confidence intervals for adjusted variables predicting survival at discharge is shown in Supplementary Fig. S3.

**Table 2:** Multivariable logistic regression analysis for favorable neurological outcome and survival at discharge.

	Favorable neurological outcome			Survival at discharge		
Variables	OR	95 % CI	*P*-value	OR	95 % CI	*P*-value
Age (per one year)	0.97	0.95, 0.99	<0.001	0.97	0.95, 0.99	0.001
Male	0.41	0.23, 0.74	0.004	0.56	0.25, 1.17	0.137
Witnessed cardiac arrest	0.78	0.43, 1.42	0.410	0.79	0.39, 1.55	0.509
Bystander cardiopulmonary resuscitation	1.92	1.19, 3.14	0.008	1.44	0.82, 2.52	0.198
Transient ROSC	1.89	1.01, 3.55	0.047	4.42	1.93, 11.3	<0.001
Estimated low flow time (per one minute)	1.00	0.99, 1.01	0.794	0.99	0.98, 1.00	0.026
Shockable cardiac rhythm on arrest	1.00	0.56, 1.79	0.996	1.09	0.57, 2.04	0.790
Shockable cardiac rhythm on hospital arrival	1.71	0.93, 3.17	0.088	2.46	1.21, 5.06	0.013
Shockable cardiac rhythm at ECMO initiation	1.21	0.67, 2.18	0.526	1.15	0.59, 2.23	0.672
Clusters	−	−	−	−	−	−
Normal range (reference)	−	−	−	−	−	−
Corrected hyponatremia	1.30	0.74, 2.29	0.368	1.11	0.56, 2.25	0.771
Overcorrected	0.76	0.35, 1.59	0.467	0.33	0.15, 0.73	0.006
High trend	1.29	0.75, 2.24	0.362	0.96	0.50, 1.88	0.910

OR, odds ratio; CI, confidence interval; ROSC, return of spontaneous circulation; ECMO, extracorporeal membrane oxygenation.

### Sensitivity analyses

Two sensitivity analyses were conducted to evaluate the robustness of our findings. First, when analyzing patients with death exclusion limited to within 2 days of ICU admission (*n* = 442), no significant differences in neurological outcomes were observed across sodium trajectory clusters, while the association between the OC cluster and reduced survival remained significant (OR: 0.30, 95 % CI: 0.14–0.64, *p* = 0.002) (Supplementary Table S2). Second, multiple imputation analysis demonstrated consistent results, with no significant association between the OC cluster and neurological outcomes (OR: 0.56, 95 % CI: 0.29–1.07, *p* = 0.083), while the OC cluster showed significantly lower odds of survival (OR: 0.30, 95 % CI: 0.15–0.58, *p* < 0.001) (Supplementary Table S3). Both sensitivity analyses confirmed the robustness of our primary findings regarding the lack of association between sodium trajectories and neurological outcomes, and the consistent association between sodium overcorrection and decreased survival, independent of the exclusion criteria or missing data handling approach.

## Discussion

This study identified four distinct serum sodium trajectories in OHCA patients receiving ECPR. While the multivariable analysis did not establish a statistically significant correlation between neurological outcomes and serum sodium trajectories, it did reveal that these trajectories were independently associated with survival rates. The identification of these different sodium trajectories provides important pathophysiological insights into postcardiac arrest care.

### Serum sodium trajectories and neurological outcomes

We found no significant associations between sodium correction patterns and neurological outcomes. There are several possible explanations for this finding. First, survival bias must be considered. Given that neurological outcome assessment is limited to survivors, the most severely ill patients died early and were excluded from the neurological outcome analysis. This might have led to an underestimation of the associations between sodium trajectories and neurological outcomes. Second, the timing of assessment should be considered. In this study, neurological outcomes were evaluated at 30 days, but brain recovery may continue over a longer period.^19^, ^20^ Additionally, sodium fluctuations may impact long-term neurological function recovery,^21^, ^22^ which might not be detectable in a short-term 30-day assessment.

### Serum sodium trajectories and survival

On the basis of the findings of this study suggesting an association between serum sodium trajectories and survival, several pathophysiological mechanisms may be considered. The ECMO circuit necessitates blood contact with non-endothelialized artificial surfaces, which activate the innate immune system, triggering inflammatory responses and coagulation cascades.^23^ This systemic inflammatory response involves pro-inflammatory cytokine production,^24^, ^25^, ^26^ complement activation,^27^, ^28^, ^29^ and leukocyte activation.^30^, ^31^ These observations suggest that patients are more susceptible to ECMO-induced inflammation under systemic inflammatory conditions after cardiac arrest.

In contrast, hypernatremia induces hyperosmotic stress in tissues, which has been directly linked to enhanced inflammatory responses. Under hypernatremic conditions, activation of the NFAT5 transcription factor pathway triggers the release of proinflammatory cytokines.^32^, ^33^ The association between serum hyperosmolality and increased mortality in clinical settings suggests that hypernatremia serves as a direct contributor to pathological inflammatory processes, potentially exacerbating underlying disease states.^34^

The bidirectional relationship between osmotic disturbances and inflammation may explain why patients with sodium abnormalities experience worse outcomes during ECMO therapy. ECMO-induced systemic inflammation may exacerbate hypernatremia, while hypernatremia itself amplifies inflammatory cascades. This mutually reinforcing cycle of inflammation potentially contributes to increased mortality in patients requiring extracorporeal support.

### Methodological considerations and cluster validity

Our clustering approach was designed to test the specific hypothesis that temporal sodium patterns during BBB vulnerability would affect neurological recovery in either direction—with sustained hyponatremia potentially worsening outcomes and mild hypernatremia potentially providing neuroprotection. The four identified clusters demonstrated differences in sodium trajectory characteristics, validating our classification approach and capturing the full spectrum of sodium trajectories from corrected hyponatremia to persistent hypernatremia.

### Comparison with previous studies

Our findings confirm and extend previous research on sodium abnormalities in post-cardiac arrest patients. While earlier studies demonstrated associations between sodium abnormalities and poor outcomes using single time-point measurements,^6^, ^7^ our trajectory-based approach during the BBB vulnerability period^10^ revealed that the pattern of sodium correction, rather than absolute levels alone, may be critical for clinical outcomes. The association between overcorrection to hypernatremia and decreased survival aligns with previous research on cellular dehydration effects^8^ but contrasts with our hypothesis of potential neuroprotective benefits. Importantly, despite experimental evidence that hyponatremia increases BBB permeability and contributes to cerebral edema,^12^ we found no significant association between sodium trajectories and neurological outcomes, suggesting that other factors may predominate in determining neurological recovery in ECPR patients. To our knowledge, this study represents the first trajectory-based analysis to examine sodium patterns during the critical 72-h post-arrest period in ECPR patients, thereby providing actionable clinical guidance.

### Clinical implications

Although we did not find a significant association between sodium correction patterns and neurological outcomes, our finding that overcorrection of sodium levels is associated with worse survival outcomes supports the hypothesis that sodium management is particularly important in the ECMO setting. Although further research is needed to determine whether optimized sodium management could improve neurological outcomes, strategies that avoid rapid correction and aim for gradual, stable sodium concentration progression are recommended for potentially improving survival.

### Limitations

This study has several important limitations that should be acknowledged. First, the retrospective, registry-based design limits our ability to establish causal relationships between sodium trajectories and outcomes. The single-country setting may limit the generalizability of our findings to other healthcare systems with different ECPR protocols, post-arrest care standards, and patient populations. The substantial number of exclusions introduces potential selection bias as patients with complete sodium measurements may represent a subset with more intensive monitoring or different clinical characteristics. This high exclusion rate limits the external validity of our findings. The major limitation of our study is the inability to distinguish between deliberate therapeutic interventions and spontaneous physiological changes affecting sodium levels. Various interventions, including hypertonic saline, renal replacement therapy, and fluid management strategies, might affect sodium levels and outcomes differently. Unmeasured confounding due to institutional variations in ECPR protocols, post-arrest care practices, and sodium-modulating treatments across the 36 participating centers may also have influenced our results. The registry design precluded standardization of these critical care components. Sodium fluctuations may actually be markers of underlying pathologies such as diabetes insipidus, syndrome of inappropriate secretion of antidiuretic hormone, or cerebral salt wasting, or they may indicate illness severity rather than directly contributing to outcomes.^35^, ^36^ The lack of detailed data on interventions affecting sodium levels limits our ability to interpret causality.

Additionally, our sample size limited us to four sodium trajectory clusters compared to the five patterns identified in larger general hospital populations.^37^ This constraint may have reduced our ability to detect more nuanced trajectory patterns, particularly those involving persistent hypernatremia or completely uncorrected hyponatremia.

## Conclusion

We found no significant associations between sodium correction patterns and neurological outcomes. While the overcorrection pattern was independently associated with significantly lower survival rates, sodium management emerged as a potentially modifiable factor in postcardiac arrest care. Future studies may better elucidate whether optimized sodium management could also impact neurological recovery in survivors.

## Consent for publication

Not applicable.

## Availability of data and material

The datasets analyzed during the current study are available from the corresponding author upon reasonable request.

## Funding source

None.

## CRediT authorship contribution statement

**Yu Amemiya:** Writing – original draft, Visualization, Methodology, Formal analysis, Conceptualization. **Ryo Hisamune:** Writing – review & editing, Conceptualization. **Kazuma Yamakawa:** Writing – review & editing, Conceptualization. **Ryosuke Zushi:** Supervision, Conceptualization. **Hitoshi Kobata:** Supervision, Conceptualization. **Akihiko Inoue:** Resources, Project administration, Data curation. **Toru Hifumi:** Resources, Project administration, Data curation. **Tetsuya Sakamoto:** Resources, Project administration, Data curation. **Yasuhiro Kuroda:** Resources, Project administration, Data curation. **Akira Takasu:** Supervision, Conceptualization. **Hirotaka Sawano:** . **Yuko Egawa:** . **Shunichi Kato:** . **Naofumi Bunya:** . **Takehiko Kasai:** . **Shinichi Ijuin:** . **Shinichi Nakayama:** . **Jun Kanda:** . **Seiya Kanou:** . **Toru Takiguchi:** . **Shoji Yokobori:** . **Hiroaki Takada:** . **Kazushige Inoue:** . **Ichiro Takeuchi:** . **Hiroshi Honzawa:** . **Makoto Kobayashi:** . **Tomohiro Hamagami:** . **Wataru Takayama:** . **Yasuhiro Otomo:** . **Kunihiko Maekawa:** . **Takafumi Shimizu:** . **Satoshi Nara:** . **Michitaka Nasu:** . **Kuniko Takahashi:** . **Yoshihiro Hagiwara:** . **Shigeki Kushimoto:** . **Reo Fukuda:** . **Takayuki Ogura:** . **Shin-ichiro Shiraishi:** . **Hiroshi Okamoto:** . **Norio Otani:** . **Migaku Kikuchi:** . **Kazuhiro Watanabe:** . **Takuo Nakagami:** . **Tomohisa Shoko:** . **Nobuya Kitamura:** . **Takayuki Otani:** . **Yoshinori Matsuoka:** . **Makoto Aoki:** . **Masaaki Sakuraya:** . **Hideki Arimoto:** . **Koichiro Homma:** . **Hiromichi Naito:** . **Shunichiro Nakao:** . **Jun Kunikata:** . **Hideto Yokoi:** . **Tomoya Okazaki:** . **Yoshio Tahara:** .

## Ethics approval and consent to participate

The SAVE-J II study was approved by the Clinical Research Ethics Committee of Kagawa University (Approval Number: 2018-110) as well as by the ethics committees of all participating institutions. Owing to the retrospective nature of the study, the requirement for informed consent was waived at all participating facilities.

## Declaration of competing interest

The authors declare that they have no known competing financial interests or personal relationships that could have appeared to influence the work reported in this paper.
