# Supplementary material for: Serum sodium trajectories impact clinical outcomes in extracorporeal resuscitation of cardiac arrest: A multicenter retrospective cohort study
Source: Resusc Plus. 2025 Aug 22;26:101074. doi: 10.1016/j.resplu.2025.101074 (PMC12444628; doi:10.1016/j.resplu.2025.101074)
Supplement: Supplementary Data 1 [file mmc1.docx]

**Supplementary information**

**Random forest based imputation methods**

We employed a stepwise random forest-based imputation strategy using the missForest algorithm to handle missing values in our dataset. This approach was chosen for its ability to handle mixed-type data and capture non-linear relationships between variables.

Imputation process

1. Initial data preparation

- Numerical variables were isolated from the dataset

- Variables were selected based on clinical relevance, including:

- Basic demographics (age, body weight)

- Vital signs (heart rate, blood pressure)

- Laboratory values (blood gases, electrolytes)

- Clinical scores (modified SOFA scores of renal function)

- Fluid balance parameters

2. Stepwise imputation strategy

The imputation was performed in a temporal sequence to maintain the logical progression of clinical data:

a) Baseline variables

- Basic demographic data and initial vital signs were imputed first

- Parameters: maxiter = 10, ntree = 100

b) Day 0 (at hospital arrival) values

- Initial laboratory values including:

- Serum electrolytes (Na, K, Cl)

- Creatinine

- Blood gas parameters

c) Sequential daily values (Days 1-4)

- For each subsequent day, imputation included:

- Current day's electrolyte values

- Fluid balance parameters

- Modified SOFA scores of renal function

- Previous days' values as predictors

- This approach ensured temporal consistency in the imputed values

3. Derived variables creation

Post-imputation, we calculated:

- Daily fluid balance

- Cumulative fluid balance

- 72-hour total balance

**Imputation quality assessment**

The quality of multiple imputation was assessed through four complementary approaches. First, distributional preservation was evaluated by comparing summary statistics (means, standard deviations, medians) and density distributions between original complete case data and imputed datasets. Second, correlation structure preservation was assessed by comparing correlation matrices before and after imputation, with particular focus on sodium trajectory variables. Third, plausibility checks examined whether imputed values fell within physiologically acceptable ranges, specifically evaluating sodium measurements against established normal limits (135-145 mEq/L). Fourth, the extent of missing data and patterns of missingness were characterized to ensure imputation was performed within acceptable thresholds. Quality metrics included mean distributional changes, standard deviation ratios, correlation differences, and proportions of imputed values within normal physiological ranges.

1. Missing Data Summary

Analysis focused on 8 key variables with missing data out of 820 total observations.

| Variable | Missing Count | Total N | Missing % |
| --- | --- | --- | --- |
| Sodium at hospital arrival | 21 | 820 | 2.56 |
| Sodium on ICU day 3 | 59 | 820 | 7.20 |
| Sodium on ICU day 2 | 60 | 820 | 7.32 |
| Sodium on ICU day 4 | 70 | 820 | 8.54 |
| Arterial blood gas lactate at hospital arrival, mmol/L | 77 | 820 | 9.39 |
| Sodium at ICU admission | 122 | 820 | 14.88 |
| Arterial blood gas lactate at ICU enter, mmol/L | 148 | 820 | 18.05 |

2. Imputation Quality Metrics

Summary of distribution preservation, sample sizes, and plausibility checks:

Plausibility checks were performed for sodium measurements using physiological ranges (135-145 mEq/L). Other variables were not assessed due to either absence of missing values (age) or lack of universally applicable normal ranges in the ECMO patient population.

| Variable | Original Sample | Imputed Sample | Values Imputed n (%) | Mean Change | SD Ratio | Plausible Range % |
| --- | --- | --- | --- | --- | --- | --- |
| Age, year | 820 | 820 | 0 (0%) | 0.00 | 1.00 | N/A |
| Sodium at hospital arrival | 799 | 820 | 21 (2.6%) | -0.01 | 0.99 | 76.8% |
| Sodium at ICU admission | 698 | 820 | 122 (14.9%) | 0.01 | 0.94 | 74% |
| Sodium on ICU day 2 | 760 | 820 | 60 (7.3%) | -0.01 | 0.97 | 75.4% |
| Sodium on ICU day 3 | 761 | 820 | 59 (7.2%) | -0.06 | 0.97 | 75.6% |
| Sodium on ICU day 4 | 750 | 820 | 70 (8.5%) | -0.01 | 0.98 | 73.4% |
| Lactate at ICU enter, mmol/L | 672 | 820 | 148 (18%) | -0.02 | 0.92 | N/A |

3. Correlation Structure Preservation

Sodium trajectory correlation analysis: Maximum absolute difference = 0.022 , Mean absolute difference = 0.007

4. Quality Assessment Interpretation

• Missing data rates varied from 2.6% to 18.0% across analysis variables

• Distribution preservation was excellent with mean changes ≤0.06

• Correlation structure was well preserved (max difference 0.022)

• Plausibility checks showed 75% of imputed sodium values within normal ranges

5. Distribution comparison of original and imputed data


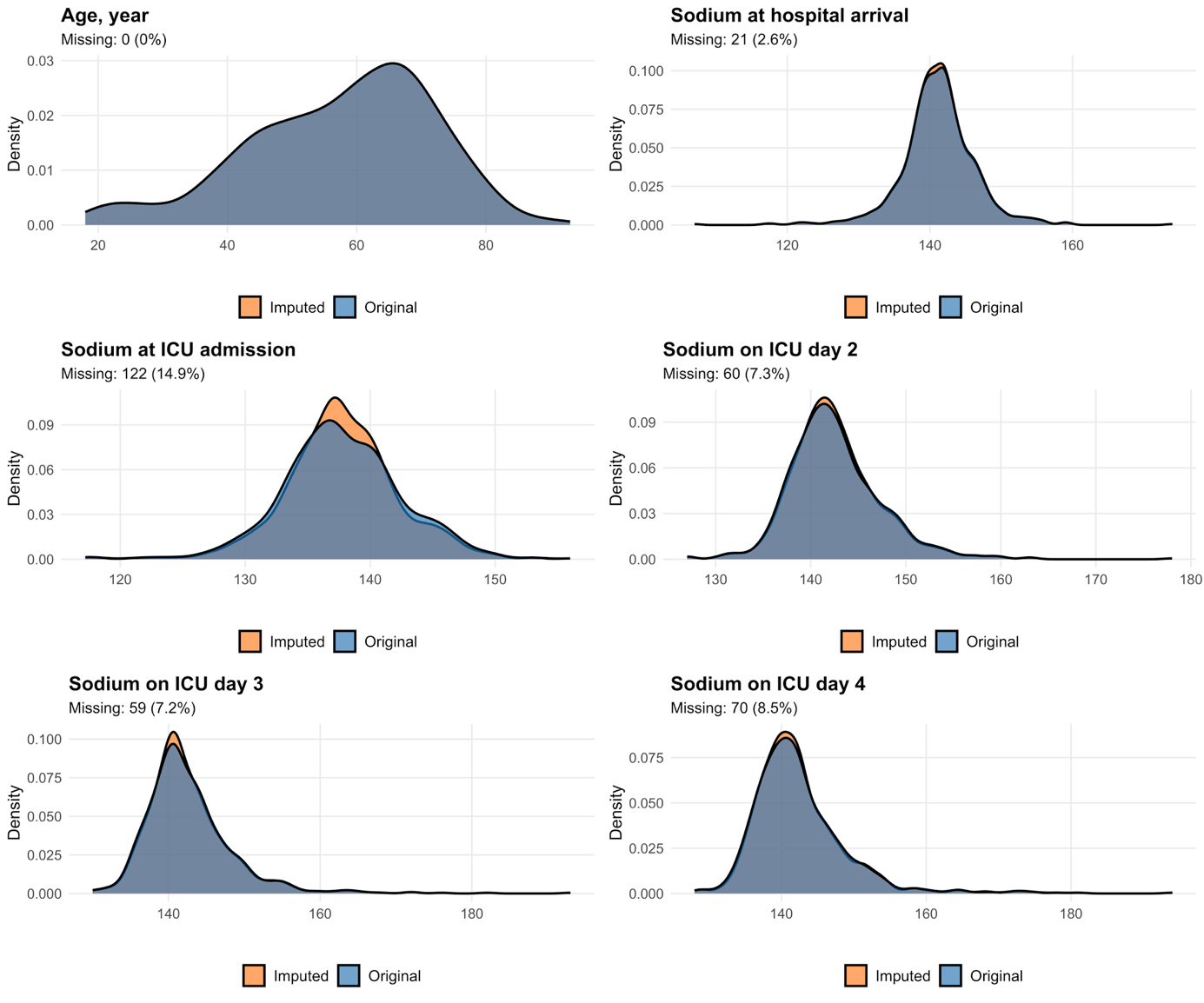


Density plots comparing original complete data (blue) and multiply imputed data (orange) for key variables. Missing rates ranged from 0% (age) to 14.9% (sodium at ICU admission). Close distribution alignment demonstrates excellent preservation of data characteristics with minimal bias introduction. Sodium measurements represent the clinical timeline from hospital arrival through ICU day 4.

| **Supplementary Tables**  **Table S1. Serum Sodium Features by Cluster** | | | | |
| --- | --- | --- | --- | --- |
| **Features** | **NR (N = 152)** | **CH (N = 96)** | **OC (N = 48)** | **HT (N = 104)** |
| Serum sodium, mEq/L | - | - | - | - |
| On hospital arrival | 142.0 [3.5] | 136.0 [4.2] | 139.7 [5.6] | 144.0 [4.4] |
| At ICU admission | 137.6 [3.3] | 133.4 [4.2] | 139.0 [4.5] | 141.1 [3.5] |
| Day 2 | 140.4 [2.8] | 139.5 [3.3] | 147.2 [4.7] | 145.1 [2.6] |
| Day 3 | 139.1 [2.5] | 140.4 [2.6] | 150.3 [4.1] | 144.6 [2.4] |
| Day 4 | 138.0 [2.5] | 140.6 [2.7] | 151.0 [4.6] | 144.4 [2.6] |
| Slope | -0.6 [0.8] | 1.6 [0.9] | 3.4 [1.4] | 0.4 [1.0] |
| Range | 7.1 [3.5] | 9.4 [3.9] | 15.6 [5.5] | 7.3 [3.1] |
| Mean | 139.4 [1.8] | 138.0 [2.5] | 145.5 [3.2] | 143.8 [2.1] |
| Median | 139.4 [1.9] | 138.8 [2.8] | 146.8 [4.1] | 144.1 [2.1] |
| Min | 135.9 [2.2] | 132.4 [3.8] | 136.9 [4.8] | 140.0 [2.7] |
| Max | 143.0 [3.3] | 141.8 [2.6] | 152.5 [4.4] | 147.3 [3.1] |
| NR, normal-range cluster; CH, corrected-hyponatremia cluster; OC, overcorrected cluster; HT, high-trend cluster. | | | | |

| **Table S2. Multivariable Logistic Regression Analysis with Death Exclusion Limited to 2 Days** | | | | | | |
| --- | --- | --- | --- | --- | --- | --- |
|  | **Favorable**  **neurological outcome** | | | **Survival at discharge** | | |
| **Variables** | **OR** | **95% CI** | **P-value** | **OR** | **95% CI** | **P-value** |
| Age (per one year) | 0.97 | 0.96, 0.99 | <0.001 | 0.97 | 0.95, 0.99 | 0.003 |
| Male | 0.44 | 0.24, 0.78 | 0.005 | 0.62 | 0.29, 1.24 | 0.193 |
| Witnessed cardiac arrest | 0.75 | 0.42, 1.35 | 0.329 | 0.74 | 0.38, 1.40 | 0.365 |
| Bystander cardiopulmonary resuscitation | 2.01 | 1.26, 3.25 | 0.004 | 1.58 | 0.93, 2.67 | 0.087 |
| Transient ROSC | 1.92 | 1.03, 3.58 | 0.040 | 4.34 | 1.97, 10.5 | <0.001 |
| Estimated low flow time (per one minute) | 1.00 | 0.99, 1.01 | 0.758 | 0.99 | 0.98, 1.00 | 0.036 |
| Shockable cardiac rhythm on arrest | 0.92 | 0.52, 1.64 | 0.786 | 0.92 | 0.50, 1.67 | 0.780 |
| Shockable cardiac rhythm on hospital arrival | 1.87 | 1.04, 3.41 | 0.039 | 2.93 | 1.52, 5.73 | 0.001 |
| Shockable cardiac rhythm at ECMO initiation | 1.24 | 0.70, 2.21 | 0.462 | 1.16 | 0.62, 2.16 | 0.632 |
| Clusters | - | - | - | - | - | - |
| Normal range (reference) | - | - | - | - | - | - |
| Corrected hyponatremia | 1.05 | 0.60, 1.84 | 0.866 | 1.00 | 0.52, 1.95 | 0.988 |
| Overcorrected | 0.65 | 0.31, 1.34 | 0.251 | 0.30 | 0.14, 0.64 | 0.002 |
| High trend | 1.15 | 0.67, 1.98 | 0.601 | 0.91 | 0.49, 1.72 | 0.778 |
| OR, odds ratio; CI, confidence interval; ROSC, return of spontaneous circulation; ECMO, extracorporeal membrane oxygenation. | | | | | | |

| **Table S3. Multivariable Logistic Regression Analysis after Multiple Imputation** | | | | | | |
| --- | --- | --- | --- | --- | --- | --- |
|  | **Favorable**  **neurological outcome** | | | **Survival at discharge** | | |
| **Variables** | **OR** | **95% CI** | **P-value** | **OR** | **95% CI** | **P-value** |
| Age (per one year) | 0.98 | 0.97, 1.00 | 0.008 | 0.98 | 0.96, 1.00 | 0.014 |
| Male | 0.39 | 0.23, 0.64 | <0.001 | 0.50 | 0.25, 0.93 | 0.035 |
| Witnessed cardiac arrest | 0.79 | 0.49, 1.28 | 0.342 | 0.92 | 0.53, 1.59 | 0.771 |
| Bystander cardiopulmonary resuscitation | 2.09 | 1.40, 3.15 | <0.001 | 1.21 | 0.75, 1.93 | 0.427 |
| Transient ROSC | 2.17 | 1.29, 3.68 | 0.004 | 4.77 | 2.26, 11.2 | <0.001 |
| Estimated low flow time (per one minute) | 1.00 | 0.99, 1.01 | 0.952 | 0.99 | 0.98, 1.00 | 0.009 |
| Shockable cardiac rhythm on arrest | 1.40 | 0.84, 2.33 | 0.199 | 1.26 | 0.72, 2.17 | 0.415 |
| Shockable cardiac rhythm on hospital arrival | 1.16 | 0.70, 1.92 | 0.574 | 1.61 | 0.89, 2.90 | 0.114 |
| Shockable cardiac rhythm at ECMO initiation | 1.67 | 1.01, 2.77 | 0.045 | 1.77 | 1.01, 3.10 | 0.045 |
| Clusters | - | - | - | - | - | - |
| Normal range (reference) | - | - | - | - | - | - |
| Corrected hyponatremia | 1.26 | 0.77, 2.05 | 0.357 | 1.01 | 0.56, 1.87 | 0.962 |
| Overcorrected | 0.56 | 0.29, 1.07 | 0.083 | 0.30 | 0.15, 0.58 | <0.001 |
| High trend | 1.23 | 0.77, 1.96 | 0.388 | 0.82 | 0.46, 1.44 | 0.485 |
| OR, odds ratio; CI, confidence interval; ROSC, return of spontaneous circulation; ECMO, extracorporeal membrane oxygenation. | | | | | | |

**Supplementary Figures**

**Figure S1. K-means clustering plot**

**
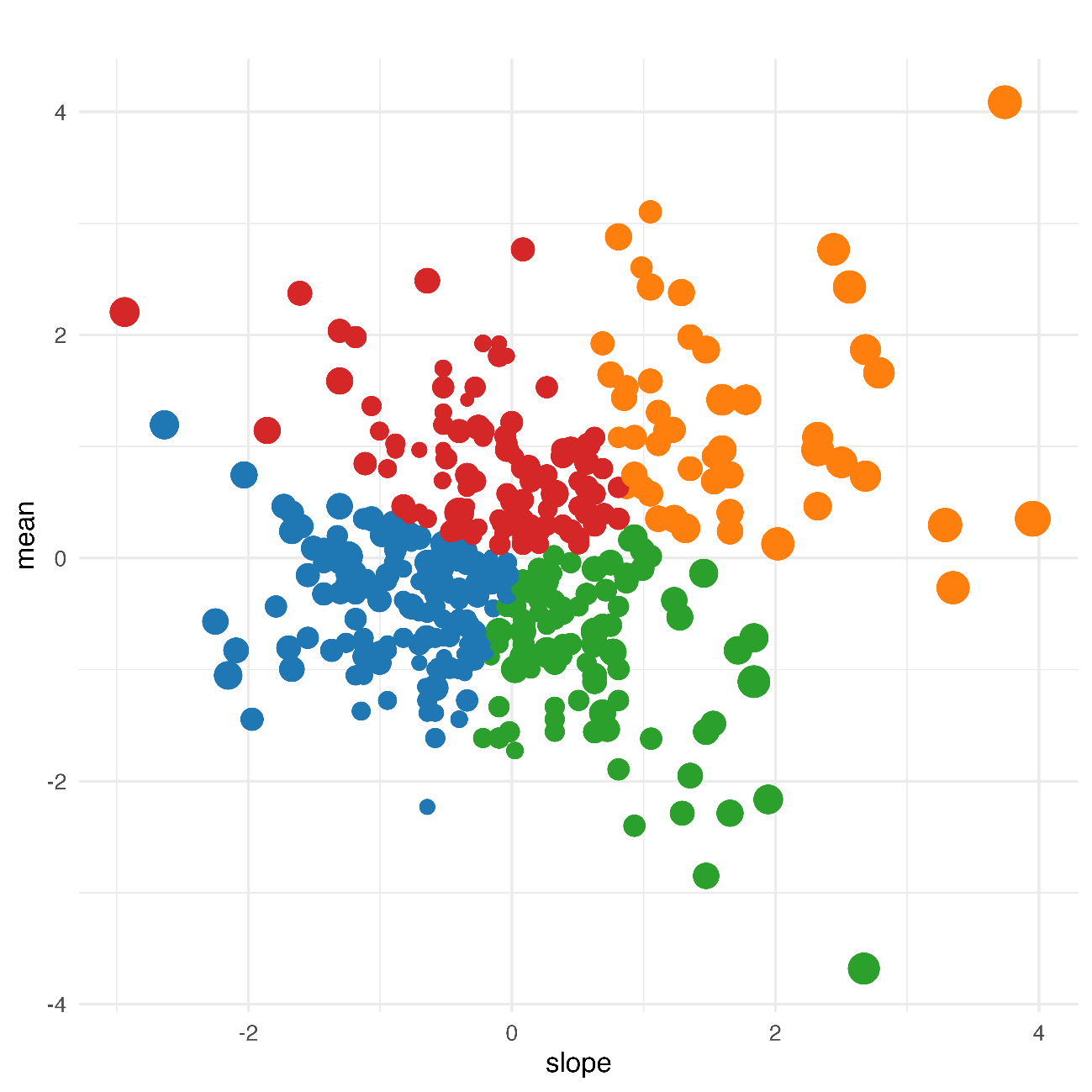
**

K-means clustering results for sodium level trajectories. Each point represents a patient plotted in two-dimensional space: slope of sodium change (x-axis) and mean serum sodium level (y-axis). Four distinct clusters are color-coded with cluster centroids marked: green = normal range; blue = corrected hyponatremia; orange = overcorrected; red = high trend. Dot size indicates the range of sodium values during the observation period.

**Figure S2. Elbow method plot**

**
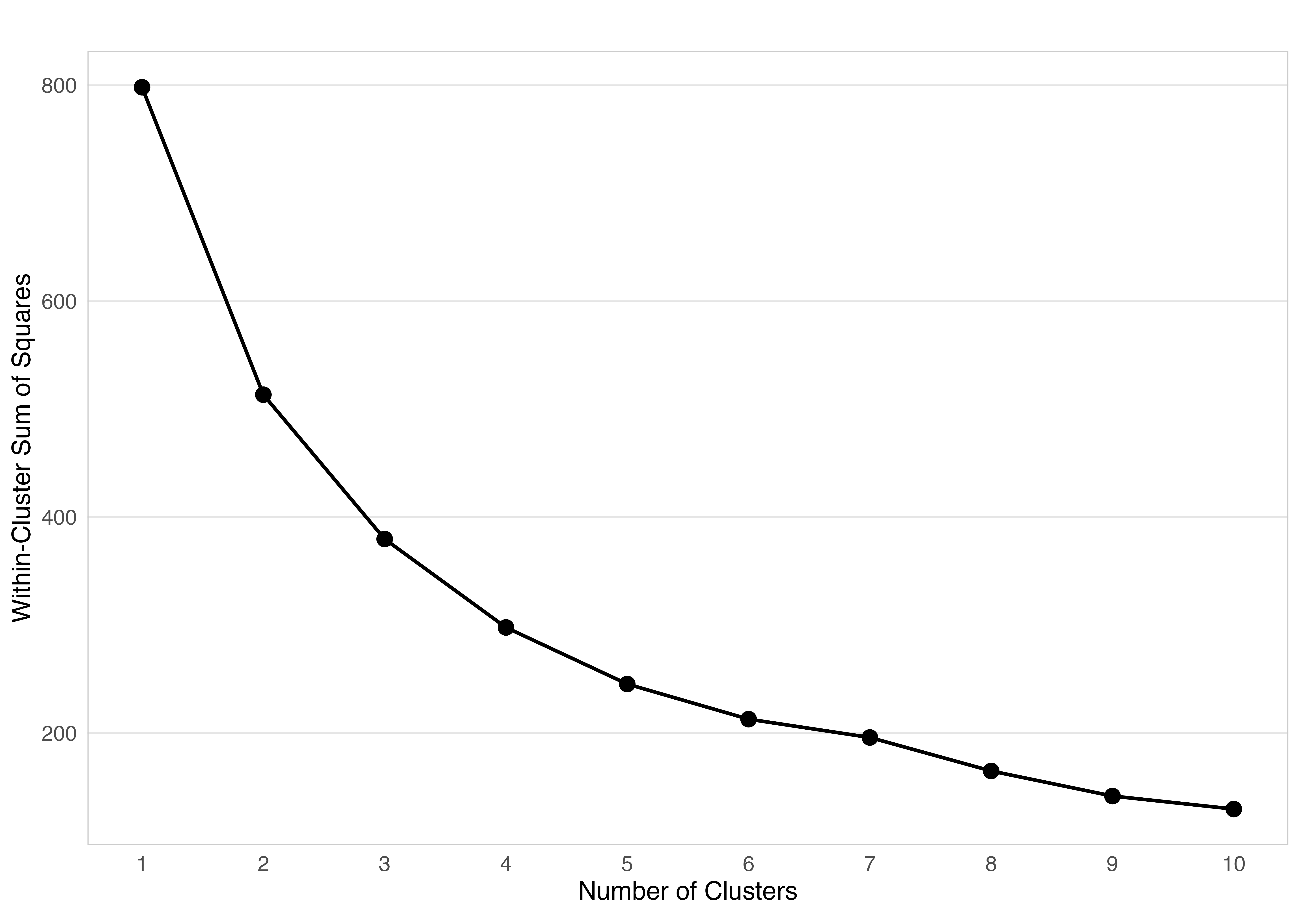
**

Determination of optimal cluster number using the elbow method. The within-cluster sum of squares is plotted against the number of clusters (k = 1–10).

**Figure S3.** Forest plot of adjusted odds ratios from multivariable logistic regression analysis predicting in-hospital survival. OR, odds ratio; CI, confidence interval.**
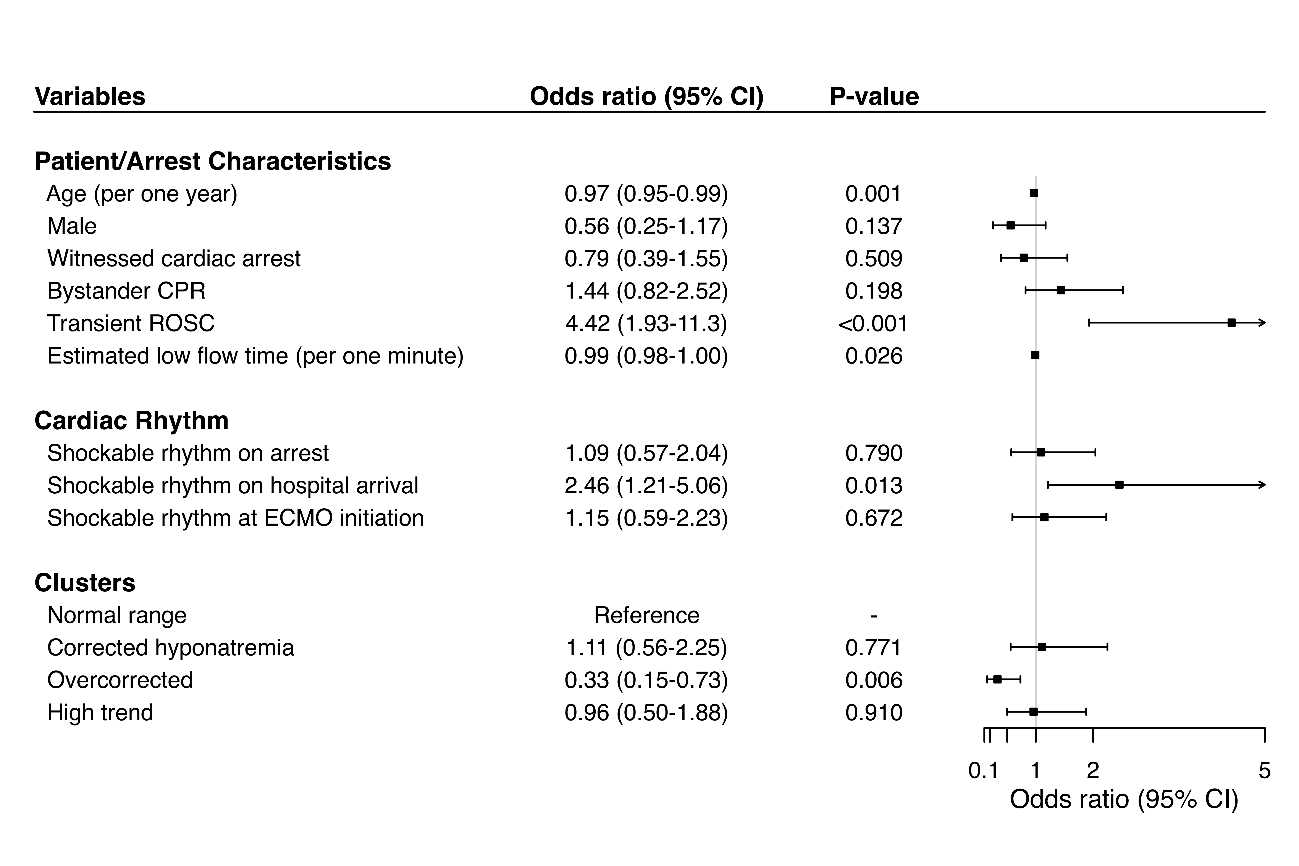
**
